# Circulating tumor DNA analysis as a real-time method for monitoring tumor burden in melanoma patients undergoing treatment with immune checkpoint blockade

**DOI:** 10.1186/s40425-014-0042-0

**Published:** 2014-12-16

**Authors:** Evan J Lipson, Victor E Velculescu, Theresa S Pritchard, Mark Sausen, Drew M Pardoll, Suzanne L Topalian, Luis A Diaz

**Affiliations:** Department of Oncology, Johns Hopkins University School of Medicine and Sidney Kimmel Comprehensive Cancer Center, Baltimore, MD USA; Department of Surgery, Johns Hopkins University School of Medicine and Sidney Kimmel Comprehensive Cancer Center, Baltimore, MD USA; Ludwig Center for Cancer Genetics and Therapeutics and the Swim Across America Laboratory, Johns Hopkins University School of Medicine and Sidney Kimmel Comprehensive Cancer Center, Baltimore, MD USA; Personal Genome Diagnostics, Baltimore, MD USA

**Keywords:** Circulating tumor DNA, Immunotherapy, Checkpoint blockade, Anti-PD-1, Ipilimumab, Biomarker

## Abstract

**Background:**

Assessment of therapeutic activity of drugs blocking immune checkpoints such as CTLA-4 and PD-1/PD-L1 can be challenging, as tumors may seem to enlarge or appear anew before regressing, due to intratumoral inflammation. We assessed whether circulating tumor DNA (ctDNA) levels could serve as an early indicator of true changes in tumor burden in patients undergoing treatment with these agents.

**Findings:**

Tumors from 12 patients with metastatic melanoma undergoing treatment with checkpoint blocking drugs were analyzed for the presence of hotspot somatic mutations in *BRAF*, *cKIT*, *NRAS*, and *TERT*. Plasma was collected serially from each patient and levels of ctDNA were compared with radiologic and clinical outcomes.

In 5 of 10 patients studied, mutations were detected in *BRAF*(1), *NRAS*(2), *TERT*(1) and *ALK*(1). Analysis of plasma from 4 of 5 patients identified mutations identical to those found in tumor specimens. Plasma ctDNA levels ranged from undetectable (<0.01%) to 5.5% of total circulating cell-free DNA. In 3 patients, increasing ctDNA levels correlated with progressive disease assessed by radiography. In one patient, ctDNA levels increased after undergoing a needle biopsy of a tumor deposit. In another patient, ctDNA levels increased initially as lymphadenopathy progressed by examination, but then became undetectable 3 weeks prior to clinical improvement.

**Conclusions:**

Levels of ctDNA correlated with clinical and radiologic outcomes, and, in one case, preceded eventual tumor regression. Further prospective analysis is required to assess the utility of ctDNA as an early biomarker of clinical outcomes in patients receiving immune checkpoint blocking drugs.

**Electronic supplementary material:**

The online version of this article (doi:10.1186/s40425-014-0042-0) contains supplementary material, which is available to authorized users.

## Findings

### Background

Our group and others have previously reported that circulating tumor DNA (ctDNA) can be detected in the plasma of patients with advanced melanoma and other malignancies [1-6]. Furthermore, levels of ctDNA can correlate with changes in tumor burden in response to surgery or chemotherapy [7,8]. The current study tested the hypothesis that ctDNA levels in the plasma of patients with metastatic melanoma could serve as early biomarkers of therapeutic responses to immune checkpoint blockade therapy.

Monoclonal antibodies blocking immune checkpoint molecules such as Programmed Death-1 (PD-1; nivolumab and pembrolizumab), PD-ligand 1 (PD-L1; BMS-936559, MPDL3280A, and MEDI4736), and Cytotoxic T lymphocyte-associated Antigen 4 (CTLA-4; ipilimumab), have mechanisms of action which differ significantly from standard cytotoxic therapies [9]. This creates challenges for assessing therapeutic activity. Checkpoint blocking drugs act directly on the immune system, rather than on the tumor, and the kinetics of tumor regression may be delayed [10,11]. In some cases, tumors assessed using conventional CT imaging criteria (RECIST; [12] appear to enlarge during therapy before regressing. In other patients, new tumors appear on therapy which later regress. Some tumors remain stable in size for a prolonged period of time, even after treatment has been stopped [13-16]. It has been proposed that apparent tumor progression may, in some cases, reflect intratumoral inflammation rather than actual tumor growth [17]. Non-invasive indicators of changes in tumor burden could provide early information about therapeutic outcomes and avoid unnecessary exposure to potentially serious immune-related toxicities in patients with true disease progression [18].

## Methods

Twelve patients with metastatic melanoma who were scheduled to receive treatment with an immune checkpoint blocking drug at our institution, including ipilimumab (anti-CTLA-4) or BMS-936559 (anti-PD-L1) (NCT00729664, Ref. [14]), provided informed consent to participate in this IRB-approved study. Archived formalin-fixed, paraffin-embedded tumor specimens were analyzed for common, recurrent somatic sequence mutations in *BRAF*, *cKIT*, *NRAS* and *TERT* [19] using standardized pyrosequencing, melting curve analysis, or Sanger sequencing techniques, as previously described [20,21]. Methodological details for the 5 patients in whom a tumor mutation was detected are included in Additional file 1: Table S1. In one case, whole exome sequencing was performed to identify a unique tumor-specific mutation that could be used to quantify ctDNA in plasma. Sample library construction, exome capture, next generation sequencing, and bioinformatic analyses were performed at Personal Genome Diagnostics (Baltimore, MD). Genomic DNA, obtained from circulating leukocytes, was fragmented and used for Illumina TruSeq library construction (Illumina, San Diego, CA). Exonic regions were captured in solution using the Agilent SureSelect 50 Mb kit (version 4) according to the manufacturer’s instructions (Agilent, Santa Clara, CA). Paired-end sequencing, resulting in 100 bases from each end of the fragments, was performed using a HiSeq 2000 Genome Analyzer System (Illumina, San Diego, CA). The sequences were aligned to the human genome reference sequence (hg18) using the Eland algorithm of CASAVA 1.7 software (Illumina, San Diego, CA). The chastity filter of the BaseCall software of Illumina was used to select sequence reads for subsequent analysis. The ELANDv2 algorithm of CASAVA 1.7 software (Illumina, San Diego, CA) was then applied to identify point mutations and small insertions and deletions. Potential somatic mutations were filtered and visually inspected as described previously [22,23].

Blood was collected in K_2_EDTA collection vials from each patient prior to therapy, and then at approximately 2-4 week intervals during treatment. Within 3 hours of collection, samples were centrifuged at 810 g for ten minutes at room temperature. In a sterile biosafety cabinet, plasma was pooled and aliquoted. Samples were centrifuged at 18,000 g for ten minutes at room temperature. The supernatant was transferred to cryovials and immediately stored at -80C. Buffy coat was collected from each EDTA tube and immediately stored at -80C. DNA was extracted using the QIAamp Circulating Nucleic Acid Kit (Qiagen, Valencia, CA). BEAMing technology (Beads, Emulsification, Amplification and Magnetics; Inostics, Hamburg, Germany) was used to detect and quantify circulating melanoma-derived DNA [7,24]. In one case, a custom PCR and next-generation sequencing approach was required due to difficulty in generating a BEAMing probe specific to the TERT promoter locus, due to high GC content. Briefly, DNA was partitioned into reactions each containing approximately 1,000 genomic equivalents (GE), from which a mutation was called if present in at least two independent PCR and next-generation sequencing reactions, as previously described [25]. ctDNA fractions were quantified by taking the total mutant observations divided by the total GE analyzed. The lower limit of sensitivity for this assay was dependent on the total GE analyzed, and ranged from 1,603 to 15,308, yielding a sensitivity of >0.1%. Results were correlated with radiologic and clinical outcomes.

## Results

Two patients died due to disease progression prior to completing their courses of therapy. Of ten evaluable patients, one demonstrated a mutation in *BRAF* (1799T > A (V600E)), none in *cKIT* and two in *NRAS* (182A > G (Q61R), 181C > A (Q61K)). *TERT* mutation testing was performed on 2 patients whose tumors were wild-type for the 3 genes listed above, and one demonstrated a mutation (Chr5: 1,295,228-9;GG > AA). In another patient, whole exome sequencing analysis of tumor and normal samples revealed a mutation in *ALK* (Chr2: 29,551,215; C > T); plasma DNA levels were not analyzed for this patient (Table 1).

**Table 1:** **Tumor**-**specific mutation analysis of 10 melanoma tumor specimens**

**ID** ^**a**^	**BRAF**	**cKIT**	**NRAS**	**Other**	**Therapy**	**Radiographic response**	**ctDNA level analyzed?**
01	WT	WT	WT	**Chr5: 1,295,228-9**	ipilimumab	Immune-related PR	Y
				**GG > AA (TERT)** ^**b**^			
03	**1799T > A**	WT	NT	NT	BMS-936559	PD	Y
05	WT	WT	WT	NT	ipilimumab	PD	N
06	WT	WT	WT	NT	BMS-936559	PD	N
07	WT	WT	WT	NT	ipilimumab	PD	N
08	WT	WT	**182A > G**	NT	BMS-936559	PD	Y
09	WT	WT	WT	NT	BMS-936559	PD	N
10	WT	WT	**181C > A**	NT	ipilimumab	PD	Y
11	WT	WT	WT	**Chr2: 29,551,215**	ipilimumab	CR	N
				**C > T (ALK)** ^**b**^			
12	WT	WT	NT^c^	NT	ipilimumab	PD	N

Archived formalin-fixed, paraffin-embedded tumor specimens were analyzed for common, recurrent somatic sequence mutations in *BRAF*, *cKIT*, *NRAS* and *TERT* [19] using standardized pyrosequencing, melting curve analysis, or Sanger sequencing techniques, as previously described (Wood et. al., Science. 2007 Nov 16;318(5853):1108-13. and Parsons et. al., Science. 2008 Sep 26;321(5897):1807-12.). No mutation was detected in 5 of 10 patients. Previously reported mutations associated with melanoma (*BRAF*, *NRAS*, *TERT*) were found in 4 patients. For one subject (#11) whose tumor was found to be wild type for each of the above genes, whole exome sequencing analysis of tumor and normal samples was employed to identify tumor-specific (somatic) sequence and copy number alterations. (WT, wild type; NT, not tested; PR, partial response; PD, progressive disease; CR, complete response; ^a^patient ID numbers are not sequential as 2 patients who died due to disease progression prior to completing their courses of therapy are not included in Table 1; ^b^genomic position, hg19; ^c^No PCR amplified product was obtained after repeated attempts).

Analysis of plasma from 4 patients identified mutations identical to those found in tumor specimens (*BRAF*, *NRAS*, *TERT*). Plasma ctDNA levels measured as a fraction of total GE in cell-free DNA ranged from below the detection limits of the PCR/next-generation sequencing assay (<0.1%) or the BEAMing assay (<0.01%) to 5.5% of total circulating DNA.

**Figure 1:**
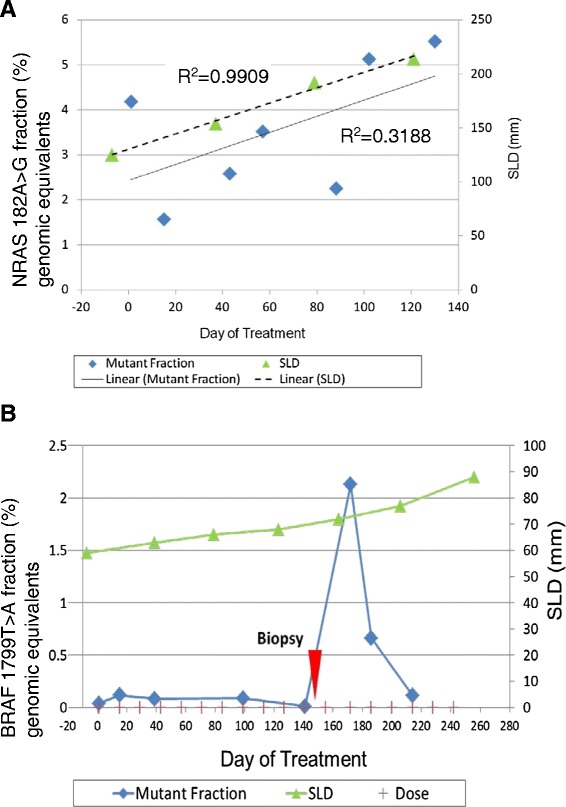
**Correlation of ctDNA measurements with clinical course. A)** Increasing levels of ctDNA (NRAS A182G) correlate with progressive disease assessed by radiography in patient #08, a 52-year-old man with metastatic melanoma who received BMS-936559 (anti-PD-L1). **B)** Levels of ctDNA (BRAF V600E) in patient #03, a 69-year-old woman with metastatic melanoma who received BMS-936559, increased substantially after a needle biopsy of a lower extremity soft tissue metastasis on treatment day 155 (red arrow). SLD, sum of longest tumor diameters.

Increasing levels of tumor-derived ctDNA were seen in conjunction with progressive disease assessed by radiography in three patients. In one patient, ctDNA levels increased substantially after a needle biopsy of a tumor deposit was performed, validating the sensitivity of these methods (Figure 1).

**Figure 2:**
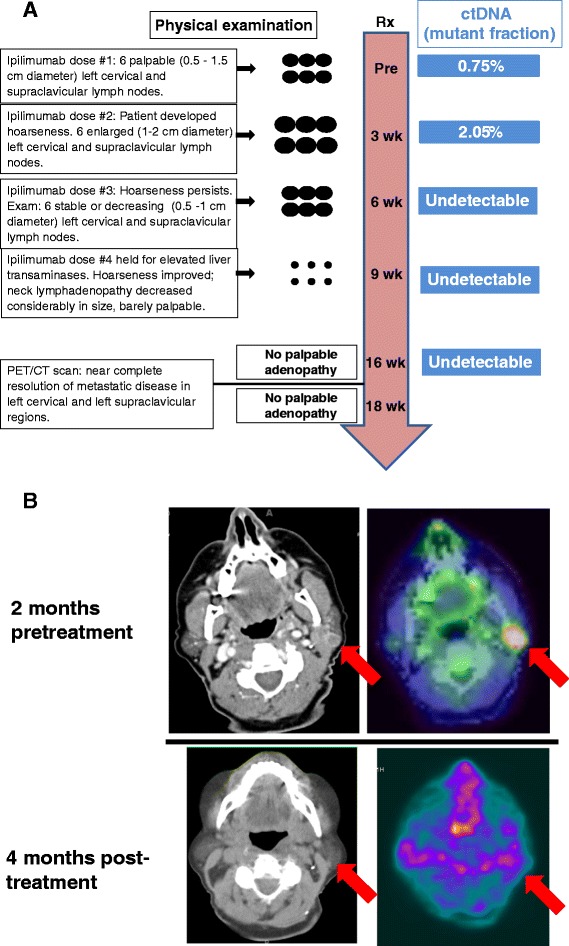
**Clinical course and ctDNA measurements for patient** **#01**, **a 68**-**year**-**old woman with biopsy**-**proven unresectable melanoma of the left neck and left supraclavicular regions.** She received ipilimumab as first-line therapy, to which she had an “immune-related” response. **A)** Treatment timeline. Ipilimumab (anti-CTLA-4, 3 mg/kg) was administered intravenously every 3 weeks for 3 doses. ctDNA levels (TERTmut) increased initially as metastatic lymph nodes enlarged on physical examination (week 3), but ctDNA became undetectable at week 6 even though metastatic lymph nodes were still palpable. Significant disease regression was noted clinically 3 weeks later and complete disease resolution was demonstrated on CT and FDG-PET scans performed 4 months after treatment initiation. **B)** CT and FDG-PET images demonstrating the resolution of palpable cervical lymphadenopathy after administration of ipilimumab.

In one patient who experienced an “immune-related” response to ipilimumab therapy -- clinical disease progression early in the treatment course, followed by a sustained response to treatment -- plasma levels of tumor-derived DNA became undetectable several weeks prior to clinical tumor regression (Figure 2).

## Discussion

Clinical management of patients undergoing therapy with immune checkpoint blocking drugs can be challenging. Radiographic changes seen on conventional CT scans can be misleading, as tumors may appear to enlarge or appear anew before later regressing. Furthermore, prolonged disease stabilization may contribute to overall survival benefits. Although serum levels of CEA, CA19-9 and PSA can be used as surrogate markers of tumor burden changes in colon, pancreas and prostate cancer, many cancers such as melanoma have no validated tumor marker. However, genetic alterations contained in human cancers, including gene deletions or amplifications, point mutations and chromosomal rearrangements, can distinguish cell-free tumor DNA from normal DNA and can serve as personalized biomarkers of disease in patients with advanced melanoma and other malignancies.

Approximately 90% of melanomas harbor a point mutation in *BRAF*, *NRAS* or *cKIT* [26]. Likewise, mutations in the *TERT* promoter have been reported in approximately 40-70% of melanoma tumors [19,27]. These high-frequency mutations could be used to track ctDNA levels in a majority of patients with metastatic melanoma. For patients with other tumor types with more diverse and/or private mutations, whole exome sequencing may be required in order to identify a genetic alteration suitable for detection in the circulation.

In this pilot study, changes in ctDNA levels correlated with physical examination and radiologic outcomes in patients receiving checkpoint blockade agents. In one case, decreasing ctDNA levels preceded eventual clinical and radiographic tumor regression. Thus, ctDNA levels may serve as an early biomarker, reflecting tumor burden changes more quickly than those detected using CT radiography. Although these findings require further prospective analysis and validation in larger numbers of patients, an easily-measured, accurate, plasma-based marker of disease such as ctDNA would potentially change patient management in several settings. First, decreasing levels of ctDNA detected during the appearance of clinical or radiographic disease progression might aid in the early identification of patients whose tumors will soon respond to treatment and, therefore, should continue on therapy. Second, ctDNA levels that are rising in the period between radiologic evaluations might prompt the clinician to consider early radiologic restaging, since rising ctDNA could indicate true disease progression, or tumor lysis preceding regression. Third, in patients whose cancers recur after a prolonged period of regression off therapy [13], ctDNA may serve as an early indicator of tumor growth and prompt a re-initiation of therapy. Lastly, in early-stage patients who have undergone surgical resection of disease, plasma evidence of residual disease may influence recommendations for adjuvant therapy [28,29].

The initial increase in levels of ctDNA in patient #01 potentially supports the theory that apparent tumor progression may, in some cases, reflect intratumoral inflammation and immune-mediated tumor destruction, rather than actual tumor growth [17]. In analogous fashion, an acute, destructive event within a tumor deposit in patient #03 (i.e., a physical disruption with a biopsy needle) appeared to significantly increase ctDNA levels. One question that stems from this observation concerns the possible seeding of distant disease by disruption of a melanoma deposit. However, several studies have demonstrated that surgical transection of a primary melanoma at initial biopsy does not affect patient survival, regardless of biopsy technique employed [30-32].

Our study is the first to demonstrate that changes in ctDNA may be predictive of the anti-tumor activity of immune checkpoint blocking drugs. Further study involving larger numbers of patients is needed to assess the utility of ctDNA levels in detecting fluctuations in tumor burden prior to clinically or radiographically measureable changes in patients receiving these agents.

## Additional file

Additional file 1: Table S1. Methods used for mutational analysis of tumor tissue for the 5 patients in our study in whom a genetic mutation was detected.

## Abbreviations

BEAM: Beads, Emulsification, Amplification and Magnetics
ctDNA: Circulating tumor DNA
CR: Complete response
CT: Computed tomography
CTLA-4: Cytotoxic T Lymphocyte Antigen-4
GE: Genomic equivalents
PR: Partial response
PCR: Polymerase chain reaction
PET: Positron emission tomography
PD-L1: Programmed Death Ligand-1
PD-1: Programmed Death-1
PD: Progressive disease
SLD: Sum of Longest Diameters
SD: Stable disease
