# Supplementary material for: Circulating tumor DNA analysis as a real-time method for monitoring tumor burden in melanoma patients undergoing treatment with immune checkpoint blockade
Source: J Immunother Cancer. 2014 Dec 16;2:42. doi: 10.1186/s40425-014-0042-0 (PMC4267741; doi:10.1186/s40425-014-0042-0)
Supplement: Additional file 1: Table S1. — Methods used for mutational analysis of tumor tissue for the 5 patients in our study in whom a genetic mutation was detected. [file 40425_2014_42_MOESM1_ESM.doc]

Additional file 1: Table S1: Methods used for mutational analysis of tumor tissue for the 5 patients in our study in whom a genetic mutation was detected.

| **PATIENT ID** | **MUTATION** | **METHODS** |
| --- | --- | --- |
| 1 | Chr5: 1,295,228-9  GG>AA (TERT) | Sanger sequencing techniques performed at Personal Genome Diagnostics, Baltimore, MD. Methods as previously described:  1. Wood et. al., Science. 2007 Nov 16;318(5853):1108-13  2. Parsons et. al., Science. 2008 Sep 26;321(5897):1807-12 |
| 3 | 1799T>A (BRAF) | ResponseDX BRAF testing: A hematoxylin and eosin (H&E) stained section of a patient's formalin-fixed paraffin embedded tumor is evaluated by a board-certified pathologist for tumor content. Specific area of the specimen are identified for microdissection of tumor cells from normal cells. Adjacent sections of the tumor are sectioned and stained with nuclear fast red (NFR) for visualization for microdissection. After isolation and lysis of tumor cells, DNA is isolated from the specimen using the QiaAMP Minelute columns. Zymo-spin IV-HRC columns are used to remove potentially inhibitory melanin from the sample before the PCR step. The presence of DNA mutations is detected by PCR with primers specific for the V600E and V600K mutation ins BRAF. The results are expressed relative to the amount of PCR product of the non-mutated gene in each case. Performed at Response Genetics, Los Angeles, CA. |
| 8 | 182A>G (NRAS) | Pyrosequencing: In fixed tissue samples, the area of tumor was grossly microdissected prior to DNA extraction. Extracted genomic DNA was subjected to PCR-based sequencing of the NRAS gene in two separate reactions for exon 1 (spanning codons 12 # 13) and exon 2 (codon 61). Test Performed by Quest Diagnostics Nichols Institute, Chantilly, VA |
| 10 | 181C>A (NRAS) | Pyrosequencing: In fixed tissue samples, the area of tumor was grossly microdissected prior to DNA extraction. Extracted genomic DNA was subjected to PCR-based sequencing of the NRAS gene in two separate reactions for exon 1 (spanning codons 12 # 13) and exon 2 (codon 61). Test Performed by Quest Diagnostics Nichols Institute, Chantilly, VA |
| 11 | Chr2: 29,551,215  C>T (ALK) | Whole exome sequencing performed at Personal Genome Diagnostics, Baltimore, MD. Methods described in manuscript. |
